# Supplementary material for: Development of chemokine network inhibitors using combinatorial saturation mutagenesis
Source: Commun Biol. 2025 Apr 3;8:549. doi: 10.1038/s42003-025-07778-6 (PMC11969024; doi:10.1038/s42003-025-07778-6)
Supplement: Supplementary file 4 — Supplementary Data 2 [file 42003_2025_7778_MOESM4_ESM.zip › SupplementaryData3/scripts/xattune_dr.tar!/./script/analysis/JK/JK_E162_CXCL9_ATC_plot.pdf]

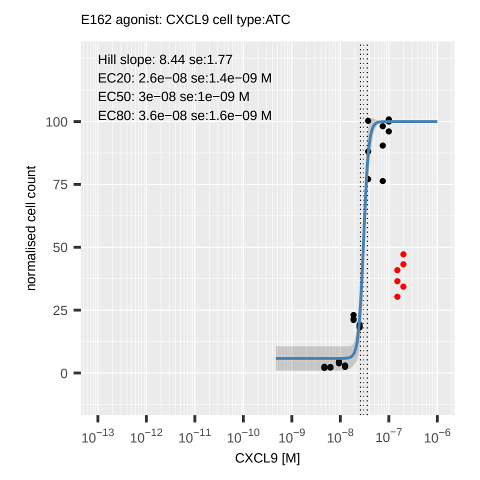

E162 agonist: CXCL9 cell type:ATC
Hill slope: 8.44 se:1.77
EC20: 2.6e−08 se:1.4e−09 M
EC50: 3e−08 se:1e−09 M
EC80: 3.6e−08 se:1.6e−09 M
normalised cell count
100
75
50
25
0
$10^{-13}$
$10^{-12}$
$10^{-11}$
$10^{-10}$
$10^{-9}$
$10^{-8}$
$10^{-7}$
$10^{-6}$
CXCL9 [M]
